# Dietary intervention in gestational diabetes: a qualitative study of the acceptability and feasibility of a novel whole-diet intervention in healthcare professionals

**DOI:** 10.1017/S0007114523001666

**Published:** 2024-01-28

**Authors:** Laura Caroline Kusinski, Rebecca Richards, Danielle L. Jones, Elizabeth Turner, Deborah J Hughes, Pamela Dyson, Amy L. Ahern, Claire Louise Meek

**Affiliations:** 1 Wellcome-Trust MRC Institute of Metabolic Science Metabolic Research Laboratories, University of Cambridge, Cambridge Biomedical Campus, Cambridge CB2 0QQ, UK; 2 MRC Epidemiology Unit, University of Cambridge, Cambridge Biomedical Campus, Cambridge CB2 0QQ, UK; 3 Cambridge Universities NHS Foundation Trust, Cambridge, Hills Road, Cambridge CB2 0QQ, UK; 4 Oxford University Hospitals NHS Foundation Trust, John Radcliffe Hospital, Headley Way, Headington, Oxford OX3 9DU, UK; 5 Oxford Centre for Diabetes, Endocrinology & Metabolism, Churchill Hospital, University of Oxford, Headington, Oxford OX3 7LE, UK

**Keywords:** Qualitative research, Food intervention, Gestational diabetes, Lifestyle, Pregnancy, Reduced calorie, Randomised control trial

## Abstract

Gestational diabetes is treated with medical nutrition therapy, delivered by healthcare professionals; however, the optimal diet for affected women is unknown. Randomised controlled trials, such as the DiGest (Dietary Intervention in Gestational Diabetes) trial, will address this knowledge gap, but the acceptability of whole-diet interventions in pregnancy is unclear. Whole-diet approaches reduce bias but require high levels of participant commitment and long intervention periods to generate meaningful clinical outcomes. We aimed to assess healthcare professionals’ views on the acceptability of the DiGest dietbox intervention for women with gestational diabetes and to identify any barriers to adherence which could be addressed to support good recruitment and retention to the DiGest trial. Female healthcare professionals (*n* 16) were randomly allocated to receive a DiGest dietbox containing 1200 or 2000 kcal/d including at least one weeks’ food. A semi-structured interview was conducted to explore participants’ experience of the intervention. Interviews were audio-recorded, transcribed verbatim and analysed thematically using NVivo software. Based on the findings of qualitative interviews, modifications were made to the dietboxes. Participants found the dietboxes convenient and enjoyed the variety and taste of the meals. Factors which facilitated adherence included participants having a good understanding of study aims and sufficient organisational skills to facilitate weekly meal planning in advance. Barriers to adherence included peer pressure during social occasions and feelings of deprivation or hunger (affecting both standard and reduced calorie groups). Healthcare professionals considered random allocation to a whole-diet replacement intervention to be acceptable and feasible in a clinical environment and offered benefits to participants including convenience.

Gestational diabetes, the most common medical complication of pregnancy, is managed with medical nutritional therapy^(1)^. However, the optimal diet in women with gestational diabetes has not been identified. A recent meta-analysis identified multiple studies addressing nutritional management of gestational diabetes^(2)^, but many were small in size or flawed in design, demonstrating a need for better methodologies for nutritional studies in pregnancy.

Randomised controlled trials are considered the gold standard of scientific evidence and are widely used to assess novel medical therapies. However, their use in the field of nutrition is controversial^(3)^. Nutritional and dietary studies pose challenges for randomisation and blinding, and long intervention periods are often needed to generate clinically meaningful outcomes^(4)^. This has led to some criticism that randomised studies are unfeasible and inappropriate for nutritional research^(3,5)^.

There are three main methods of using randomised controlled trials in nutritional research, including use of dietary advice as an intervention, use of food items or food supplements in comparison with placebo, or using ‘whole-diet’ strategies which focus on altering the quality or composition of the whole diet^(6)^. While providing individualised dietary advice is labour-intensive, it is achievable in a clinical environment and provides evidence directly applicable to real-world clinical care. However, bias due to pre-existing dietary choices, available income, cooking skills and nutritional knowledge can attenuate the positive effect of the intervention^(4)^. Food supplements, alone or in comparison with placebo, have been widely used, but results are likely to vary depending on whether or not previous dietary nutrient intake was adequate and often this is incompletely assessed^(4)^. Randomised whole-diet approaches, such as those where all food is provided to participants, reduce bias but are often considered unfeasible due to the requirement for high levels of participant commitment during the long intervention periods required to generate meaningful clinical outcomes^(4)^. However, from a behavioural perspective, whole-diet food provision can make it easier for people to adhere to a diet by simplifying dietary choices, structuring eating occasions, increasing accuracy of calorie and portion size estimation and modifying the home food environment to increase availability of nutritious food^(7)^.

Well-designed studies of whole-diet provision can provide novel data about disease aetiology and management. An example of a successful whole-diet intervention was the DiRECT trial, a reduced-energy diet (825–853 kcal/d) in non-pregnant adults with type 2 diabetes for 12–20 weeks, which resulted in improvements in glycaemia, induced diabetes remission and reduced cardiovascular risk^(8)^. Whole-diet approaches are less common in pregnancy but have still been used. For example, Hodson and colleagues recruited women with gestational diabetes who received a 4-week dietary intervention of 1200 kcal/d, which was well accepted and resulted in a significant weight loss (of 0·4 kg per week)^(9)^. These data highlight that these types of energy-restricted whole-diet interventions can be useful tools to improve clinical outcomes.

The DiGest trial (Dietary Intervention in Gestational Diabetes) is a randomised, blinded, controlled trial of a reduced-energy diet compared with a standard energy diet^(10)^. The aim of the DiGest trial is to assess if restricting energy intake improves maternal glycaemia and pregnancy outcomes, particularly infant birth weight and perinatal complications. Participants are recruited at 20–32 weeks’ gestation (usually 28–32 weeks’ gestation) and are randomised to receive a standard energy dietbox (2000 kcal/d) or a reduced energy dietbox (1200 kcal/d). Trial allocation is blinded from participants, study team and healthcare professionals. Participants continue to receive weekly dietboxes until delivery of the baby. Previous work has demonstrated that calorie restriction in pregnant women with gestational diabetes is safe^(11,12)^. We considered several options for the DiGest intervention, including a whole-diet approach or providing personalised dietary advice with macronutrient targets. We chose to use a whole-diet approach to ensure adequate nutrition even in the context of a low-energy diet in pregnancy. A whole-diet approach also reduces socio-economic or educational barriers which might influence participants’ ability to following an intervention diet with dietary advice alone.

Our initial short-term testing of feasibility was conducted in healthcare professionals caring for pregnant women with gestational diabetes. This was so that we could optimise the intervention before trialling it in women with gestational diabetes. We hypothesised that the feasibility of whole-diet interventions in pregnant women with gestational diabetes was likely to be strongly influenced by the views of healthcare professionals. Healthcare professionals have the opportunity to support adherence to the intervention but also could reduce adherence if they considered the intervention less suitable than standard care. Healthcare professionals might also be concerned with the emotional or practical burden of a whole-diet intervention to women who are under their care. The main objective of this study was to assess healthcare professionals’ views on the acceptability of the DiGest dietbox intervention for women patients with gestational diabetes. A secondary objective was to assess if the healthcare professionals identified any barriers to adherence which could be addressed to support good recruitment and retention to the DiGest trial.

## Experimental methods

The DiGest randomised controlled trial has been described fully elsewhere^(10)^. The main DiGest trial and this ancillary study were conducted according to the guidelines laid down in the Declaration of Helsinki, and all procedures involving human subjects were approved by the West Midlands Research Ethics Committee (reference 18/WM/0191; ISRCTN 65152174). This ancillary study was conducted alongside the main DiGest trial for the first 6 months of recruitment. Healthcare professionals were eligible if they were working at a research-active hospital at the time of the study and involved in the clinical care of women with gestational diabetes. Written informed consent was obtained from all participants.

### Participants

A group of non-pregnant female healthcare professionals were recruited for this study by a female postdoctoral researcher (LCK) using a convenience sampling method. Healthcare professionals including nurses, midwives, dieticians and doctors were given the opportunity to test the acceptability of the intervention over a 1-week period. Healthcare professionals at Cambridge University Hospitals NHS Foundation Trust and North West Anglia NHS Foundation Trust were approached face to face and through publicity via email. Two eligible healthcare professionals declined to the study due to illness during the first wave of the Covid-19 pandemic. Written informed consent was obtained from all participants. Healthcare professionals received a dietbox for 1 week. Healthcare professionals were excluded from the study if they had multiple dietary restrictions due to preference or allergy.

### Dietbox intervention

All participants were randomly assigned to receive a whole-diet intervention provided as a dietbox containing either 1200 or 2000 kcal/d including three meals per d and a daily snack pack. To protect blinding among healthcare professionals who may eat lunch in the same office, participants were randomised by study site. Participants made their food choices in advance on a custom-designed website. All participants were blinded to energy allocation.

The dietboxes were produced by Cape and Hartley Foods Ltd and specifically designed for women with gestational diabetes under direction from CLM and PD. Final menus were assessed for nutritional content. All meals designed to provide 40 % of energy from carbohydrate, 35 % from fat and 25 % from protein. Each daily snack pack contained a number of items which together provided 40 % of calories from carbohydrate, 35 % from fat and 25 % from protein. Further details can be obtained from the senior author upon request.

### Qualitative interviews

Semi-structured qualitative interviews were performed with healthcare professionals from January to July 2020 (for interview schedule, see online Supplementary material, S1). The interview schedule was also designed to be used in women with gestational diabetes, to ensure consistency. The main qualitative interviewer (LCK) is a postdoctoral research associate (PhD) in diabetes in pregnancy and was trained in qualitative research by RR and AA who assisted with the analysis. LCK approached potential participants directly and provided a participant information sheet. LCK explained the purpose of the study was to get an accurate assessment of the strengths and weaknesses of the DiGest dietboxes and to understand the challenges faced by the non-pregnant healthcare professionals in adhering to the dietboxes. LCK explained all information would be anonymous and would not be available to their colleagues or other DiGest investigators in identifiable form. All potential participants were given at least 48 h to read the participant information sheet and reflect prior to consent being received.

Based on our previous work within this field^(13)^ (Kusinski *et al.*, manuscript submitted), we anticipated that between 15 and 20 interviews would provide sufficient richness of data to develop our understanding of the experiences of the healthcare professionals using the dietboxes. A single interviewer conducted all interviews. When they considered that no new information was coming from additional interviews, they conducted a preliminary analysis to verify data saturation before stopping recruitment.

Topic guides were developed after informal discussions with healthcare professionals and women with gestational diabetes and pilot-tested during the first six interviews where performance was satisfactory. No further changes were required to the topic guide. Questions aimed to assess both the physical aspects of the intervention and study, for example, healthcare professionals’ views on the taste and suitability of the food, and also the emotional implications of participating in this type of study and how it impacted their daily lives. Interviews were conducted face to face at their workplace in a private room. All interviews were audio-recorded. Additional field notes were taken by LCK to address any discrepancy during transcription. Interviews ranged from 25 to 45 min (average 4755 words) and were audio-recorded and transcribed verbatim by a commercial company under an appropriate confidentiality agreement. No repeat interviews were conducted. Transcripts were available to participants for comment and correction if required. One of the sixteen participants chose to read the interview transcript, but no further comments to the original transcript were made.

### Qualitative analysis

An inductive, thematic analysis was conducted using NVivo (QSR International version 12 Pro)^(14)^. A thematic approach was used as it helps to provide insights by moving from a broad reading of early-phase data to conceptualisation of codes and themes, followed by their interpretation^(14)^. Each transcript was read several times by LCK for familiarity, noting meanings and patterns. Initial codes were generated by LCK from line-by-line scrutiny of each data item, and mind maps were created to identify the links between codes and possible overarching themes. A subsample of transcripts (*n* 2) were double-coded using the same method, independently by a second researcher (RR) with experience in qualitative research. Double-coding of the data from RR was conducted to help minimise biased interpretations of the data and aid reflexivity when coding. Codes were discussed by LCK and RR and refined and amended via an iterative process. Next, codes were organised into meaningful subthemes and main overarching themes which captured the essence of the codes associated with it. Themes were reviewed and refined by reviewing each data item within a theme to ensure coherence and the resulting thematic framework was reviewed by a third researcher (AA).

Both researchers (LCK and RR) maintained an awareness of how their own personal characteristics and values may have influenced data collection and/or analysis. LCK is a postdoctoral researcher with experience in the effect of nutrition upon fetal programming and placental health. She has previously undertaken another qualitative study in this field (manuscript submitted to press). LCK was already known to all participants prior to recruitment for the qualitative study. Participants were aware that LCK is a member of the DiGest team but not directly involved in the content or planning of the dietboxes and not involved in the clinical care of women with diabetes in pregnancy.

The second researcher (RR) is a postdoctoral researcher and Health Psychologist with experience in conducting qualitative interviews and analysis. RR does not have experience of gestational diabetes so may not fully understand the views of the healthcare professionals and their opinions on how the dietboxes might work for women with GDM and, however, is interested and experienced in dietary interventions for weight loss.

## Results

After sixteen participants were interviewed, we were no longer identifying new themes within the data and thus considered that data saturation had been achieved. Baseline characteristics of the healthcare professionals who took part in this study are shown in Table 1.

**Table 1.** Baseline characteristics of healthcare professional participants. Data are presented as median (range)

	Healthcare professionals *n* 16	
	Median	Range
Age (years)	44·4	29·9–60·7
BMI (kg/m^2^)	25·0	19·0–32·8
Ethnicity	13/16 European ethnicity	81·2 %
Profession	Midwife (4 standard calorie/3 reduced calorie) Nurse (3 standard calorie/1 reduced calorie) Doctor/consultant (3 standard calorie/1 reduced calorie) Dietician (0 standard calorie/1 reduced calorie)	

**Fig. 1.**
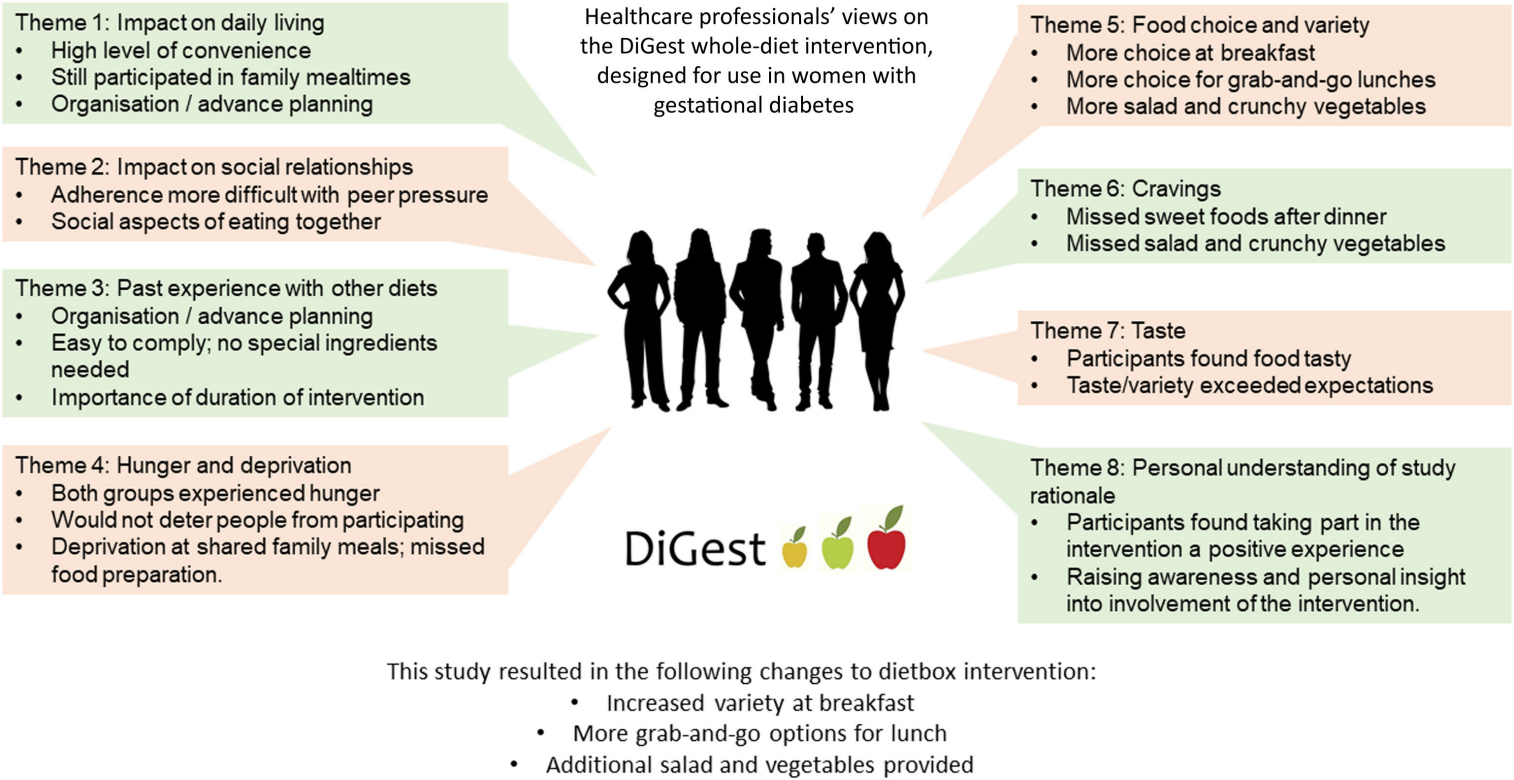
Summary of themes identified in the qualitative study, and actions taken to improve the acceptability of the dietboxes prior to use in pregnant women with gestational diabetes.

From the interviews, eight key themes were identified (Fig. 1): (1) impact on daily living, (2) impact on social relationships, (3) past experience with other diets, (4) hunger and deprivation, (5) food choice and variety, (6) cravings, (7) taste, and (8) personal understanding of study rationale. Direct quotations from participants for each theme are given in Table 2.

**Table 2.** Quotations from participants for each theme

Theme	Quotations from healthcare professionals *n* 16
*Impact on daily living*	*‘… it was more convenient I think. Normally I would have something leftover that I will bring for the lunch or I’ll go and buy something from canteen, but having this with me that means I always had food with me and brought snacks as well with me, so it was actually easier that way. I didn’t have to worry about what am I going to take tomorrow, or if I didn’t have time in-between my morning and afternoon clinic for example, then I knew that there was food with me so I could quickly eat that. So that was actually very convenient’. ID36* *‘Yeah, still, generally ate with the rest of the family, anyway, and just ate my different portion. And, although my boys found it quite amusing, actually there were a couple of reasonable conversations about food and nutrition, that they started. They soon got bored once I got into it. But they, you know, they did ask questions, it’s quite a good question prompt. And when I reflected back on it, I thought, actually, for some women who take part in DiGest, maybe actually, again, it would be a good prompt for other members of the family to learn more or maybe think about making their own dietary changes’. ID24* *‘As my children are grown up and they knew that I take part it wasn’t a problem, it was just they enjoyed the meal I cooked for them’. ID34* *‘So I liked that there were options that you could tailor to feel like you were having a meal with your family because it was very similar to what we would eat, which I thought would make me much less likely to cheat if I was doing it longer term’. ID26*
*Impact on social relationships*	‘*We’ve got big, extended family and, again, we kind of take turns having roast dinner at somebody’s house. And it just so happened it was my turn, so it’s a big family having a big shared meal. And the food is quite part of it, and the sharing food and the kind of serving each other, passing stuff up and down the table. And, yeah, I did feel a little bit miffed then that, that altered my experience of normal kind of Sunday dinner. And that, again, it was the social aspect of it, not actually what was on my plate, although that was different to everyone else. It was the social, yeah’. ID24* *‘The only two times, so when [laughs] when I did cheat and not stick, and roast dinner. It was social things that made it hard, it wasn’t because I didn’t like the food or it wasn’t enough or it didn’t taste good enough. It was, yeah, it was social things. That might just be sort of like my family’s dynamic, whenever we socialise there’s food’. ID24* *‘Well goodness I’m doing this for 10 weeks, there’s that thing about a lot of the socialising we do in this country is around food and I was potentially going to be out with a neighbour and we were talking about either going out for a cup of coffee in the afternoon and sort of afternoon tea type thing or having friends round for dinner or meeting up with people, so I could see that as 10 weeks potentially again being quite tricky for people’. ID37* *‘I think there would be more of a social pressure from people that you’re with to have whatever they’re having, so I think it’s just that social aspect’. ID37*
*Past experience with other diets*	*‘I think, especially when there’s someone, an organised plan, I think when you’re trying to do it yourself it’s very difficult to be compliant, but when it’s an organised, pre-prepared plan like Slimming World is, just thinking before I did this, DiGest one, yeah, then I think I am compliant, yeah’. ID38* *‘If things are difficult, in terms of oh you’ve got to get the right ingredients and I’ve not got that and I’ve not done the shopping and, you know, and then you think oh, you’re kind of veer off it a bit. So yeah. So it’s easy to drop off if the circumstances are not right’. ID25* ‘*I’ve tried many times to diet for weight loss. Calorie counting, low carb, that sort of thing’. ID45* *‘I am if I’m in the right sort of frame of mind to do it, I definitely am’. ID30*
*Hunger and deprivation*	*‘Yeah, the snack packs were loads in the bag. I couldn’t think about how anybody could eat all that amount of food in one day’. ID25* *‘I think there was a couple of days where in the evening I didn’t manage to finish all of my meal but again it would vary’. ID40* *‘I never felt hungry. The only other time that I felt like perhaps I, oh, I wish I wasn’t doing this today, was on a Sunday. …. And, yeah, I did feel a little bit miffed then that….altered my experience of normal kind of Sunday dinner’. ID24* *‘Missing cooking, for me that was hard, so I think interestingly when you’re recruiting women it’s helpful to kind of gauge their experience with food, for me I personally love food, so the experience of making a meal is part of my experience of eating a meal, like I enjoy them equally, so that that for me was, yeah, the hardest part probably was just feeling like I couldn’t do what I would normally do, in the experience of making food’. ID28* *‘I feel like I’ve gone on about the hunger but I never got to the point where I was just like ‘that is it’. I would have got used to it. Probably just I’m a glutton’. ID26* *‘I think it was because I was hungry that it was so hard, so I tried every day to not eat anything else, and I just, you know, sometimes it would just be like that I’d just eat my lunch super early, because I had all my meals when I was in the working day, I had them here, and other days I just was hungry in-between, so once I’d eaten all of my snacks I then was reaching for something else. That was the hunger was the main thing, I think just I obviously eat more than that would have normally allowed me’. ID28*
*Food choice and variety*	*‘I think that although there was a really good variety of food for me to choose from, and I’m not fussy and I like spicy and I eat meat, so there was quite a good range for me to pick from’. ID24* *‘So like the vegetarian bean chilli, I never would have kind of cooked that for myself so that was good, yeah, it was nice having a bit, trying things I wouldn’t normally try’. ID23* *‘I think if you’re the sort of person who eats salads and things or does a bit of a side salad or a couple of portions of vegetables with whatever you’re cooking for dinner, and you’ve either steamed them or boiled them or done something that is fresh. And maybe it’s more of a visual thing, having it very visual on your plate. But, yes, I did, I missed, more salady stuff than vegetables, but that’s probably just because that’s what I do at home’. ID24* *‘I did think, when I had my lasagne, I was like, ‘Oh, I need a little side salad’. And know it’s all there, like, in my head, like a little bit of green, like fresh food would have probably made it a little bit nicer as well’. ID30* *‘I don’t know how it works with the study to say those kind of vegetables raw or steamed you can add because it doesn’t have an effect on the blood sugars and as well those kind of like salad, lettuce, cucumber, whatever, if it can’t be provided because that is the thing which I missed’. ID34*
*Cravings*	*‘In the evening you do feel like something sweet, which you don’t have a great deal in the snack packs which is sweet. I mean there was some chocolate, which was great’. ID45* *‘Yeah, I think I’m a sweet toothed person, unfortunately. And if I’d have used, see, now I know, if I’d used, if I did it again I would choose the snack pack that had that chocolate rice cake in every day, that snack pack’. ID38* *‘I’m a real vegetable girl, so I craved vegetables, and so I didn’t, yeah, I didn’t even feel I could count one portion of a five a day from vegetables at all, from any of my meals’. ID28* *‘But to be fair during the day and especially a working day never tempted to go and get anything else at all, because I’ve normally got all the snacks with me as well, so there was always something to go to, even if it was very small’. ID41*
*Taste*	*‘I don’t tend to eat ready meals, and I wouldn’t buy a ready meal as something like a turkey dinner. So I was feeling a little bit dubious and very hard done by that I was cooking these roast dinners for everybody else. And, actually, it was alright, I was quite pleasantly surprised’. ID24* *‘Spiced seeds in the snack pack, they were really nice. I looked at this little bag, and thought, really [laughs]? I think this looks like something I feed my chickens. And they tasted amazing, so, yeah’. ID24* *‘I enjoyed overall taking part. And I really found the food tasty, like the omelette with sag aloo, I really want that again. [Laughter] And the, yeah, the wraps as well. I think it was the spiced Moroccan chicken wrap, yeah, I’ve just written yum next to that one. Really liked that. Like I’m kind of sad that I can’t just go into Marks & Spencer’s and buy that again*’. *ID26*
*Personal understanding of study rationale*	***‘**I think it’s been a really good experience, actually, to be able to, because we are going to spend a lot of time with these ladies. And, if we are able to tell them from personal experience, I think that brings quite a lot of reassurance for them’. ID24* *‘All the conversations that it started. It’s been, I don’t know any other study or trial recently, that I can think of off the top of my head, that’s gotten a team talking so much. So the team here of research nurses, midwives, our admin, I don’t know another study that’s gotten everyone talking so much’. ID24* *‘I felt that it would give me much more insight into what the women are going to be doing and that when talking about the study if women had fears about the food and what it was going to be like and that would have potentially prohibited them taking part, that it would be good to be able to say that I had done it and explained what it’s like, you know. I felt I would be better prepared to answer their questions on the study participation’. ID26* *‘I thought, well if I’m going to have to try and, not sell it to people but, you know, support people going through it, I mean have women understand, give them a positive view of how, what the experience is like and things, I thought it was important I had that understanding really of what the experience was like’. ID27* *‘I think mainly because we’re seeing patients who are on the study, so for me to know what their experience would actually look like, it helps me when they’re asking questions for me to be able to say, I’ve tried it, I can tell you something about it’. ID28*

### Theme 1: impact on daily living

Most participants reported how the dietary intervention influenced their day-to-day lives, and this was particularly relevant in the workplace. On the whole, participants found the convenience of having pre-prepared food, which they could easily take to work, to be a substantial benefit of taking part in the study.

Convenience was also mentioned in the home setting. Although most participants had a family to cook for, they did not feel the food negatively impacted family mealtimes, despite them having to cook an alternative meal for their families.

Another key feature of the dietboxes was that they were able to cater for similar types of meals to those usually eaten by participants. Many participants felt that it was not a huge change to their daily eating habits, and they could very much feel part of family mealtimes even though they were eating different meals.

An important subtheme within this theme is organisation. Many participants mentioned that planning meals enabled them to reduce the impact upon their daily lives. For example, if a participant has ordered meals for every day that week that need to be heated up in a microwave, this will not work well at a workplace with no access to this facility.

*‘Having a hot meal at work is easy enough for us because we’re not too far from a kitchen. Although you kind of have to choose your time carefully because any time after about five past twelve there’s a queue of people waiting to use the one microwave’. ID24*

*‘I mean the actual bringing them into work, the heating them up in the microwave at work, that was super easy, you know, that wasn’t difficult, that didn’t require much of me at all’. ID28*

The menu has some ‘grab-and-go’ lunch options such as wraps to facilitate more flexibility, but this was not always considered at the point of food ordering. Overall, the dietboxes did not seem to disrupt the day-to-day eating habits of the participants and most enjoyed the convenience the dietboxes provided.

*‘I just had two of the wraps. I had them for lunch because actually I was, I was pleased because, like I say, I’d forgotten it was half-term and we did go out a couple of days and we were out all day, you know, so I was able to take them with me’. ID40*

### Theme 2: impact on social relationships

Participants identified one of the hardest aspects of taking part in the study was the impact it had on their social relationships, mainly outside the home. Food is a crucial part of many social occasions.

*‘The only time I cheated. It was a big cheat because we had a Chinese takeaway. And that was based, really, on the social aspect of the evening rather than the food’. ID24*

It was at such occasions that participants found themselves more likely to deviate from the plan, mainly due to the social pressures of other people. The DiGest menu includes options like roast dinners or takeaway meals that allow participants to choose to match what their fellow diners are eating. However, this does not eliminate the pressure from others to eat what they are eating.

It is important to note that participants suggested that if they were receiving the dietboxes for a longer period of time (as a DiGest participant would be), these social interactions would likely be the most common time that they would eat additional food. The healthcare professionals only used the dietboxes for a week, but they felt that doing this longer term would have a big impact on their social life and relationships.

### Theme 3: past experience with other diets

This theme was not relevant for all participants as not all had been exposed to other types of diets. For those that had, many referred to some key points. Many participants mentioned that having a structured food regimen was key to adherence to a new diet and considered the dietboxes to help with this as they fit easily into their lifestyle. Some participants mentioned that features of other diets, such as ensuring all the correct ingredients were available to make a meal, meant that they would not be able to follow the necessary plan, and this would often lead to deviating from the diet and adherence being compromised.

One of the most crucial aspects to adherence, in light of previous diets, is knowing the length of time required for the dietary intervention. It was highlighted that when there is a definite timeline for how the intervention lasts, it provides a clearer structure for the participant and an end target.

*‘I thought it looked doable…. because I knew it was only for a week….there’s choice of food. So I think that’s fundamental, especially thinking about the ladies that will be doing it for weeks and weeks. But, even just for a week, seeing that there was quite a few options of food, and because I’m vegetarian there was that option, and I could see that on the information sheet. So that made me want to do it’. ID38*

*‘I suppose a few years ago I joined Slimming World and then didn’t continue with it….It just didn’t fit into my busy life’. ID25*

The DiGest diet is for a set period of time which is clearly set out to the participants before they start the study. Emphasising this may be an important aspect to ensure that participants will be as prepared as possible for changing their eating habits. Although all women with gestational diabetes are encouraged to maintain long-term healthy eating habits, the trial aims to assess if the dietary intervention improves perinatal outcomes.

### Theme 4: hunger and deprivation

Although participants were randomly assigned a standard energy or lower energy dietbox, hunger and/or deprivation was mentioned by both groups. Some participants in the standard calorie group considered that they did not feel hungry, but often they felt there was perhaps too much food. Participants on both arms are warned to avoid overeating and that they should eat until they are satisfied and not necessarily to finish their allocated portion size. This appeared only to be an issue raised on a standard calorie diet.

*‘I felt like I might have been on the higher calorie one because I just felt like there was a lot of food’. ID40*

Although some participants felt that there was surplus food, it was interesting to note that there were times when these participants still felt deprived but this was much more in terms of impact on lifestyle. Some participants saw this deprivation in relation to cooking and preparing food. The experience of preparing a meal was highlighted by some as a key part of the enjoyment of food, and having that aspect taken away was limiting.

Hunger itself was a key theme that was anticipated, as the lower energy dietbox is limited to 1200 kcal. Although participants were made fully aware that this might be a feature of taking part in the study, it was mentioned frequently in the interviews conducted with the participants on this arm. Although some participants found the hunger a difficult aspect, it was clear that this would not act as a deterrent. When asked if they would recommend taking part in the study to women with gestational diabetes, the consensus was that they would.

*‘I definitely would (recommend it) because I think you do get in food rut and I don’t know that, you know, when you are tired in pregnancy and that the point most of them would sign up to this they’d still be working and then they’ve got to think about potentially changing their diet quite drastically and that’s quite a hard change to make. You’ve got to invest quite a lot in your choices when you are shopping and cooking and meal planning, and I think the reassurance that I would have from that headache almost being taken off me and adjusting to how I need to eat to help would really reassure me during my pregnancy’. ID26*

*‘I think it’s really exciting, you know, I’ve really enjoyed taking part for my week, so you know. If, so yeah, if I had a baby, and gestational diabetes, I’d sign up for it…. I wouldn’t deter anybody from going on it, it’s a really positive study’. ID27*

### Theme 5: food choice and variety

Ensuring that participants felt like they were having a good variety of food choice was an important aspect when designing the dietboxes as they had to cater for a wide variety of women with different background and food habits. Most of the participants were happy with the variety of food that was provided for lunch and dinner. However, a small number of participants mentioned that the variety at breakfast was too limited. This was usually the case when a participant did not like a particular type of food such as nuts or eggs. This would limit their choices in what they could choose and so the variety in these cases was restricted.

*‘I did struggle with the breakfasts, massively, really struggled. But the rest of it, there was loads for me to pick from’. ID30*

*‘I’m not allergic to nuts but I just don’t like nuts and they pretty much all had, almost all of them had nuts in, so there was only three things that I could eat, so there wasn’t much variety’. ID23*

Most participants mentioned a perceived lack of fresh vegetables and salad in the meals and considered this a disadvantage of the dietboxes. Although each meal is nutritionally balanced and healthy, most contained cooked vegetables. Having the fresh crunch of a vegetable or salad was important to many of the participants. Some participants mentioned that there were some meals which seemed smaller than others, and having additional salad or vegetables would help making the meal more filling or attractive without adding many extra calories.

*‘I really missed the sort of crunch of something, you know. Yes, I know that nutritionally there are enough vegetables in it but a sort of crisp vegetable I missed’. ID26*

### Theme 6: cravings

When asked about if there was anything that the participants were particularly missing or craving, on the whole most of the participants were satisfied. However, cravings for something sweet, specifically after the main meal, was something that was highlighted during a number of interviews. However, those that did crave something sweet also acknowledged that the dietboxes do cater for this and found that with some forward planning they could save something from the snack pack that was sweeter, such as fruit or the dark-chocolate-covered rice cake.

*‘I don’t know if it’s possible to maybe put something sweet in there as a dessert kind of option, I don’t know, but I did get a bit of a craving for something sweet after dinner times’. ID23*

In addition to sweet foods, another craving that was mentioned was for fresh vegetables and salad as highlighted in the previous theme. Interestingly, craving vegetables is something we had not anticipated as all main meals provided contain one or two portions of vegetables, with additional fruit or vegetables in the snack packs. Some participants were unable to identify vegetables in their meals, even when having vegetarian options. However, fresh vegetables provide texture which cannot be completely replicated using frozen meals. Overall the participants felt that there was sufficient number and variety of snacks provided to address any cravings.

*‘I think I would crave for fresh home-cooked food. I think regardless of how brilliant your chef is and the taste was lovely and all the quality point of view everything is lovely, but you still crave to cook fresh yourself and eat food’. ID36*

### Theme 7: taste

It was clear that some of the healthcare professionals had pre-conceived ideas of how the food would taste and approached eating ‘ready meals’ with apprehension. This also was the case with the snack packs, where participants were already making assumptions about how some snacks would taste before trying it. The majority of the participants were clearly satisfied with the taste of the food. There were occasions when they did not enjoy certain types of foods, and this was to be expected as personal preference is a key part of dietary choice in everyday life. It was clear that after having a week trying the food, the participants were able to identify the foods they did not like.

*‘I wasn’t really a fan of what was in the wraps, some of the wraps, so that was my only issue, I didn’t like them very much’. ID27*

*‘One day I didn’t like two of the meals and because I was hungry I ate something different’. ID29*

The feedback for taste of the food was very positive, which suggests that although the participants were eating an energy-restricted diet, they did not have to compromise on food variety and taste which is very important for the DiGest study.

### Theme 8: personal understanding of study rationale

Most participants wanted to take part in the study to gain an understanding of how the study works so that they could provide information and reassurance to potential DiGest participants during recruitment. Healthcare workers were very positive about this aspect and noted that most clinical trials do not provide an opportunity for research staff and healthcare workers to try out a trial intervention.

*‘It’s been a really good experience… I wish it was something that we could replicate with lots of studies, actually, to take part in something to this kind of level that we actually have, and appreciation of what it is to be involved’. ID24*

It has also provided a platform for the healthcare professionals to discuss the study with other members of staff and raise awareness of the study throughout the hospital. This personal insight into what is required of participants taking part in the DiGest study and sharing this experience with colleagues has been invaluable and a unique way to assess the intervention.

*‘I thought it was a fantastic study and I knew we were going to get involved from the patients’ perspective…., I just leapt at the chance because I thought actually I could do with losing a few pounds as well and just see how it is for women that come into the study’. ID25*

### Increasing satisfaction with dietbox intervention

Overall participants enjoyed the food but experienced feelings of hunger and deprivation during the study. They were also concerned about reduced participation in family food-related social events.

In response to the qualitative findings described above, several changes were made to the dietboxes in order to promote greater adherence and flexibility. The options at breakfast were increased (unsweetened porridge in a wider variety of flavours, more egg options, low-sugar granola and yogurt-based smoothies) and more ‘grab-and-go’ options for lunch were provided (e.g. wraps and other meals which could be eaten straight after defrosting). We also added a vegetable or salad pack to each dietbox to provide the texture and taste of raw food and to enhance the visual appeal of meals, for example, by allowing participants to assemble a side salad.

## Discussion

This study identified that a whole-diet intervention using dietboxes designed for women with gestational diabetes was considered feasible, acceptable and convenient to a group of female healthcare professionals involved in their care. Convenience both inside and outside the home was considered a particular benefit. Challenges to adherence included peer pressure during social occasions, feelings of deprivation or hunger and perceived insufficient raw or fresh vegetables. In response to these findings, the DiGest dietbox composition was adapted, and more fresh vegetables and salad were included. This qualitative assessment demonstrates the value of assessing healthcare professionals’ views on the suitability of a novel intervention in order to optimise the intervention prior to use in patients.

### Strengths and limitations of this study

This study assessed the acceptability of a whole-diet intervention in healthcare professionals to see if this would be achievable to use in a motivated population of pregnant women in a free-living environment. Having a target population of healthcare professionals who are involved either directly or indirectly with pregnant women with gestational diabetes was an advantage as their knowledge of what dietary expectations and restrictions are placed on these pregnant women gave them more of an insight into which aspects of the dietbox would be positive and which would be negative.

Assessment of healthcare professionals’ opinions provided information about how a non-pregnant population viewed the whole-diet intervention and perceived suitability for the target population of women with gestational diabetes. However, it is a limitation of in this study that we did not interview the target population, who may have different attitudes on the suitability of the food or potential benefits of the study to their children’s health. This was not done because this was a brief intervention for a 1-week period and offering this to the pregnant women with gestational diabetes for such a short time would have been inconvenient as they are often trying to establish a new diet regimen as part of their new diagnosis. Women with gestational diabetes will be interviewed at a later date as part of the DiGest trial, where we will compare participants’ experiences of the 1200 kcal and the 2000 kcal boxes. Interpretation of this data is not possible until the trial is complete and unblinded.

Some of the healthcare professionals did not always consider the acceptability of the boxes for pregnant women, and some of their opinions were based how the dietboxes fit into the context of their own lives. It is important to note that their views on the acceptability of the dietbox for pregnant women may differ from the acceptability of the boxes for non-pregnant healthcare professionals. Healthcare professionals only received the dietbox for 1 week, while DiGest recruits receive dietboxes for at least 8 weeks, until delivery of the baby. This different time period creates different expectations. Receiving food for only 1 week also means that if a participant did not like a particular food, then this may have influenced their overall impressions of the intervention. It is anticipated that choosing the foods for personal preference is likely to be a learning process where participants on the dietboxes for a longer period choose a range of foods and reorder the meals they find most appealing.

The healthcare professionals also had different reasons for participating in the study. As they were not pregnant and did not have gestational diabetes, they did not have the same perceived need for dietary modification to improve their health or that of a fetus. This was evident from some comments from healthcare participants, for example, regarding a desire for more sweet snacks after dinner. Pregnant women with gestational diabetes are advised to avoid sugary foods, and the lack of availability of sweet snacks was therefore a limitation imposed by the disease, not the dietboxes *per se*. It is likely that women with gestational diabetes have more to gain by adhering to the dietbox and may be more motivated to avoid adverse consequences if they chose to deviate from the food provided.

This study was designed to assess healthcare professionals’ views on the acceptability of the DiGest intervention. We relied upon self-reported satisfaction and responses to qualitative questions. Other measures, such as measuring the proportion of dietbox food not consumed, were not considered for this study but might have provided additional information. Randomisation of healthcare professionals by site was designed to reduce bias but may have influenced the study results.

### Implications of the study

Pregnancy is a time when short-term dietary change and weight change can result in meaningful differences in clinical outcomes^(1)^. We have previously shown that women who maintain a stable weight after diagnosis of gestational diabetes have improved perinatal outcomes and reduced offspring birth weight compared with women who gain weight over the same period^(15)^. In women with gestational diabetes, dietary modification to control glycaemia and weight is a fundamental aspect of management of the disease^(1)^ and has also been extensively investigated for disease prevention using a variety of different study designs. For example, two studies assessed if dietary and lifestyle counselling sessions could reduce gestational diabetes risk but yielded conflicting results^(16,17)^. Studies adding dietary components have also been performed. One recent study showed benefits from increasing olive oil and nut intake, but as there was no control of dietary carbohydrate quantity, the impact of the results upon clinical care guidelines are not straightforward^(18)^. Conversely, relatively few studies have addressed dietary choices after diagnosis of gestational diabetes. Standard dietary management of gestational diabetes involves recommendations to choose low glycaemic index foods, avoid high glycaemic index foods and encourage increased intake of nutrient-rich foods such as vegetables. A recent meta-analysis identified eighteen studies (*n* 12–152 women in each) which had assessed dietary interventions in women with gestational diabetes^(2)^. No clear benefit was identified from most individual strategies, including the low glycaemic index diet, but overall, dietary strategies were associated with a significant reduction in infant birth weight. These findings suggest that dietary strategies may be beneficial, but further specific evidence is needed to improve recommendations for clinical care. The authors also noted a high degree of heterogeneity in determining the control diet in gestational diabetes dietary studies^(19)^, suggesting that there is a wide variety in standard care for women with gestational diabetes internationally.

The current study suggests that a whole-diet intervention using a dietbox is a feasible, acceptable and convenient method of dietary control. Overall, the healthcare professionals who took part in this study were very positive about the intervention and appreciated the convenience, variety, composition and taste of the food in the DiGest dietboxes. Data from this qualitative study have highlighted that there are three main barriers to adherence to the dietbox intervention, including hunger or deprivation, influence of social aspects and length of time on the study. Awareness of barriers to adherence allows participants and research staff to have realistic expectations, to develop strategies to avoid or address feelings of hunger or deprivation or to counteract peer pressure during social events.

### Conclusions

This study is the first to explore the views of healthcare professionals on the feasibility, acceptability and composition of a blinded, randomised, controlled whole-diet intervention for pregnant women with gestational diabetes. The participants highlighted key themes that were important in optimising adherence and barriers to adherence including hunger, deprivation and peer pressure during social occasions. Healthcare professionals considered that this whole-diet approach to dietary research was likely to be acceptable and achievable in a motivated population, especially where there is perceived potential for measurable benefit in relevant clinical outcomes.

## Supplementary material

For supplementary material accompanying this paper visit https://doi.org/10.1017/S0007114523001666. click here to view supplementary material
