# Supplementary material for: Dietary intervention in gestational diabetes: a qualitative study of the acceptability and feasibility of a novel whole-diet intervention in healthcare professionals
Source: Br J Nutr. 2023 Aug 10;131(2):219–28. doi: 10.1017/S0007114523001666 (PMC10751936; doi:10.1017/S0007114523001666)
Supplement: Supplementary file 1 [file S0007114523001666sup001.docx]

**Supplementary material**

**S1: Semi-structured Interview schedule for qualitative assessment of DiGest dietboxes.**

The aim is to enable participants to share experiences about participating in the dietbox study and to identify which factors positively or negatively impacted their participation.

Participant ID _______________________ Interviewer _____________________

Location _______________________ Date _____________________

Questions:

- What other kinds of diets (slimming world, weight watchers, smartphone diet apps) have you used before?
- Why did you decide to participate in this study? How did you weigh up the pros and cons of joining this study?
- How did you feel about being randomised to a standard or lower calorie dietbox?
- Did the experience match your expectations before you started the study?
- Regarding your dietbox what are your specific thoughts on
  - Ordering your weekly dietbox
  - The range of meal choices (too many, too few, prefer more fresh or frozen options?)
  - The dietbox delivery logistics
  - Cooking the dietbox meals
  - The smartphone app
  - The DiGest weighing scales
- How easy was it to incorporate the dietbox meals into your everyday life, especially in terms of your
  - work routines
  - family meals
  - weekends
  - special occasions, birthdays or holidays
- How do you deal with times when you are eating with other people, such as at family events, at cafes or restaurants, or at your place of work?
- Which additional foods did you eat in addition to the dietbox? Why and when does this most commonly happen?
- What made it difficult to stick to dietbox foods? (feeling hungry, missing out, reactions of friends, family, partner)
- What would make it easier for you to stick to the dietbox foods?
- Has the support of your partner, family, friends and colleagues helped you stick to your dietbox foods?
- Has the support of your healthcare team (diabetes midwife, nurse, clinical team) helped you stick to your dietbox foods?
- Do you think you would be likely to continue with dietbox foods over a longer time frame?
- What did you enjoy most about participating in the study (eg meal plans, saving money, less weight gain?
- What did you enjoy least about participating in the study?
- Would you recommend participating in the dietbox study to pregnant women with gestational diabetes?
- Do you have any other comments to make about your dietboxes or the study in general?

Thank you for sharing your experiences which will help us improve the dietbox experience for women with diabetes in pregnancy in the future.
